# Labile disulfide bonds are common at the leucocyte cell surface

**DOI:** 10.1098/rsob.110010

**Published:** 2011-11

**Authors:** Clive Metcalfe, Peter Cresswell, Laura Ciaccia, Benjamin Thomas, A. Neil Barclay

**Affiliations:** 1 Sir William Dunn School of Pathology, University of Oxford, Oxford OX1 3RE, UK; 2 Department of Immunobiology, Howard Hughes Medical Institute, Yale University School of Medicine, 300 Cedar Street, New Haven, CT 06520-8011, USA

**Keywords:** disulfide bonds, membrane proteins, redox, leucocytes

## Abstract

Redox conditions change in events such as immune and platelet activation, and during viral infection, but the biochemical consequences are not well characterized. There is evidence that some disulfide bonds in membrane proteins are labile while others that are probably structurally important are not exposed at the protein surface. We have developed a proteomic/mass spectrometry method to screen for and identify non-structural, redox-labile disulfide bonds in leucocyte cell-surface proteins. These labile disulfide bonds are common, with several classes of proteins being identified and around 30 membrane proteins regularly identified under different reducing conditions including using enzymes such as thioredoxin. The proteins identified include integrins, receptors, transporters and cell–cell recognition proteins. In many cases, at least one cysteine residue was identified by mass spectrometry as being modified by the reduction process. In some cases, functional changes are predicted (e.g. in integrins and cytokine receptors) but the scale of molecular changes in membrane proteins observed suggests that widespread effects are likely on many different types of proteins including enzymes, adhesion proteins and transporters. The results imply that membrane protein activity is being modulated by a ‘redox regulator’ mechanism.

## 2. Introduction

Membrane proteins that reside on the cell surface of leucocytes contain many cysteine (Cys) residues that mainly exist in an oxidized redox state as disulfide bonds. Disulfide bonds covalently link regions of proteins together and have been thought to have a largely structural role, protecting membrane proteins from proteolysis and denaturation in the harsh extracellular environment, and linking individual polypeptides. Structural disulfide bonds are usually buried inside the core of a protein or protein domain such as those found in the core of the immunoglobulin (Ig) fold. These structural disulfide bonds are protected from reduction by small molecule and enzymatic reducing agents that can be present in the extracellular space.

Recently, it has become clear that there are disulfide bonds present in cell-surface proteins that are involved in regulating molecular function upon reduction to their constituent Cys residues. These disulfide bonds have been termed ‘allosteric’, ‘redox-labile’ or ‘forbidden’ disulfides, as reducing them often results in a change in protein structure, and hence function [1–3]. In order for a disulfide bond to be redox-labile, it has to be accessible to reducing agents; therefore, they are largely found at the surface of proteins. They are also generally under torsional strain, which makes them easier to reduce. A recent bioinformatics study based on solvent-accessibility and torsional strain of the disulfide bonds in cell-surface proteins found that about 7 per cent are potentially redox-labile [1,2].

Protein disulfide isomerases (PDIs) are present in the endoplasmic reticulum at high concentrations, where they are involved in protein-folding. There is, however, evidence that they can relocate to the cell surface and affect membrane proteins. The combination of ‘allosteric’ disulfide bonds and the presence of PDIs at the cell surface in unison offer a mechanism for regulating protein function through redox events. Changes in redox potential have been observed in immune responses and labile disulfide bonds have been implicated in many different biological functions. For instance, PDIs are secreted during platelet activation [4], where they reduce disulfide bonds in the αIIbβIII integrin [5], promoting thrombus formation. Antibodies that block the catalytic activity of PDI inhibit reduction of the integrin and reduce thrombus formation [6]. HIV-1 virus entry into CD4^+^ T cells proceeds via reduction of disulfide bonds in HIV envelope protein gp120 and in CD4 on the T cells, allowing fusion of the virus and the T cell. HIV uptake can be blocked with antibodies that inhibit PDI activity [7] and reagents that react with reduced Cys in gp120 [8]. Similarly, in Newcastle disease virus, entry is facilitated by PDI-reduced disulfide bonds, which then allow viral fusion, a process that again is inhibited with PDI antibodies [9]. Recently, it has been shown that human beta-defensin 1 is protective at epithelia against fungi and bacteria only after activation by reduction of its disulfide bonds [10].

Redox chemistry plays a key role in immune cell activation. Dendritic cells secrete the redox enzyme thioredoxin (TRX) during priming and activation of T cells [11]. It is thought that cell-surface disulfide bonds are reduced as there is an increase of free Cys at the cell surface after activation [12,13]. This reduction can modulate the activity of proteins during an immune response. For example, TRX can modulate the activity of CD30, a member of the tumour necrosis factor (TNF) receptor family through reduction of a disulfide bond; other TNF receptor family members were unaffected despite their high content of disulfide bonds [14]. In addition, macrophages secrete the enzymatically active precursor form of gamma interferon-inducible lysosomal thiol reductase (proGILT) when exposed to bacterial lipopolysaccharide (LPS), and the enzyme accumulates in the serum of animals injected with LPS [15,16].

These studies show that labile disulfide bonds are important in cell-activation events, but limited progress has been made in identifying the repertoire of proteins that are modified and the particular disulfide bonds within those proteins that are affected. We describe a proteomics-based method to systematically screen for membrane proteins that contain labile disulfide bonds. Mild reducing conditions comparable with those expected during immune activation were applied to a T cell clone, and the proteins with redox labile disulfide bonds were identified by differential chemical labelling, affinity enrichment and tandem mass spectrometry-based proteomics analysis. A wide range of membrane proteins was found to contain labile disulfide bonds. Application of this screening method to a model of inflammation indicated that modification of disulfide bonds is likely to be common during immune activation and that the activity of membrane proteins may be modified in these conditions.

## 3. Results

### 3.1. Identification of labile disulfide bonds on leucocyte surface proteins

**Figure 1.**
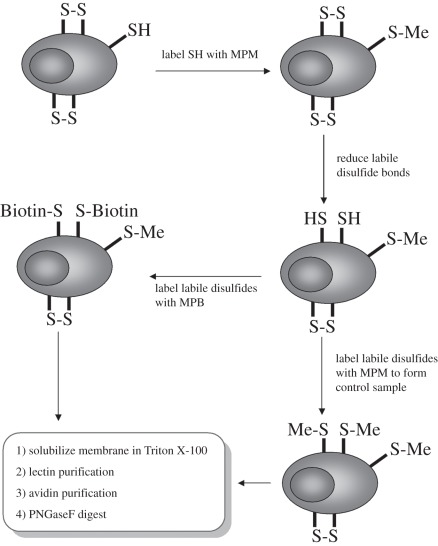
Schematic of the differential labelling strategy for labelling Cys in their different redox states. Firstly, any free Cys residues at the cell surface were blocked with MPM as indicated by S-Me. The cells were treated with one of the four reducing agents (TCEP, TRX, GILT and PDI) and labelled either with MBP (as indicated by S-Biotin) or MPM (for the control sample). The proteins with free Cys residues revealed by reduction were purified by lectin and avidin affinity chromatography, digested with trypsin and identified by mass spectrometry.

In order to screen the entire cell surface for proteins that contain redox-labile disulfide bonds, we developed a proteomics workflow based upon subjecting the cells to mild reducing conditions comparable with those expected during an immune response [11] and differentially labelling Cys residues with thiol-modifying reagents (figure 1). Methyl-PEO_12_-maleimide (MPM) was used to block any free Cys on the cell prior to reduction. Maleimide-PEO_2_-biotin (MPB), which contains a biotin moiety to enable purification of labelled proteins, was used to label any free Cys formed after mild reduction. Iodoacetamide (IAA) was used to label any Cys generated after denaturation and full reduction of the proteins prior to identification of tryptic peptides by mass spectrometry. Both MPM and MPB are cell-impermeable, ensuring that only cell-surface proteins were labelled. We used a selection of reducing agents ranging from the chemical reductant tris(2-carboxyethyl)phosphine (TCEP) to enzymatic reductants TRX, PDI and GILT [17].

The method was developed using the well-characterized mouse 2B4 T cell hybridoma (this line had also been transfected with mouse CD2 and CD244, and also expressed CD4) [18]. After labelling, cells were solubilized with non-ionic detergent and membrane glycoproteins purified by lectin affinity chromatography to reduce background in subsequent steps, followed by affinity chromatography on a monomeric avidin column to purify biotinylated surface glycoproteins. Prior to mass spectrometry analysis, *N*-linked glycans were removed from the proteins by treatment with PNGaseF and proteins were digested with trypsin. After database searching, sorting and quantitation of the data, 87 proteins were identified as candidates to contain redox-labile disulfide bonds or to be associated with proteins with labile disulfides (table 1). These proteins were either only identified in reduced cells and not controls, or they were more abundant in the reduced sample than the control based upon weighted spectral index counts (WSC).

**Table 1.** Summary of proteins identified in the screen for membrane proteins with labile disulfide bonds from the 2B4 T cell hybridoma after reduction with four reducing agents (TCEP, TRX, PDI and GILT). All the protein identifications are shown at 1% FDR relative to an empirical target decoy database and were identified with at least two unique peptide sequences.

gene	protein description	2B4 TCEP	2B4 TRX	2B4 PDI	2B4 GILT
*Adam10*	ADAM10			X	
*Adam15*	ADAM15		X	X	
*Adam17*	ADAM17		X	X	X
*Bcam*	CD239, BCAM		X		
*Bsg*	CD147, Basigin	X	X	X	
*Cd2*	CD2		X	X	X
*Cd244*	CD244, 2B4		X	X	X
*Cd27*	CD27	X			
*Cd3d*	CD3 delta		X	X	
*Cd44*	CD44	X	X	X	X
*Cd47*	CD47	X	X	X	X
*Cd96*	CD96		X	X	X
*Cd97*	CD97	X			
*Clptm1*	cleft lip and palate transmembrane protein 1 homologue		X		X
*Cr1l*	complement regulatory protein Crry			X	X
*Creld2*	cysteine-rich with EGF-like domain protein 2				X
*EG665955*	envelope glycoprotein 52			X	
*Env*	GP160	X	X	X	X
*Ephb2*	ephrin type-B receptor 2			X	
*H13*	minor histocompatibility antigen H13				x
*H2-D1*	H-2 class I histocompatibility antigen, D-K alpha chain		X	X	X
*H2-K1*	H-2 class I histocompatibility antigen, K-B alpha chain		X	X	X
*Hsp90b1*	endoplasmin	X	X	X	X
*Hspa8*	heat shock cognate 71 kDa protein		X	X	X
*Hspa9*	stress-70 protein	X	X	X	X
*Icam2*	intercellular adhesion molecule 2	X			
*Ifngr1*	CD119, interferon gamma receptor 1		X		X
*Igsf8*	CD316, immunoglobulin superfamily member 8		X	X	
*Il2rg*	CD132, cytokine receptor common subunit gamma	X^a^	X	X	
*Il6st*	CD130, interleukin-6 receptor subunit beta		X	X	X
*Itfg1*	T cell immunomodulatory protein				X
*Itga6*	integrin alpha 6	X			
*Itgal*	integrin alpha-L	X	X		
*Itgav*	integrin alpha-V	X	X		X
*Itgb1*	integrin beta-1	X	X		X
*Itgb2*	integrin beta-2	X			
*Itgb3*	integrin beta-3	X	X	X	X
*Lamp1*	lysosome-associated membrane glycoprotein 1	X			
*Lamp2*	lysosome-associated membrane glycoprotein 2		X		X
*Ldlr*	low-density lipoprotein receptor	X	X	X	X
*Lgals3bp*	galectin-3-binding protein	X	X	X	X
*Lgals8*	galectin-8		X	X	X
*Lgals9*	galectin-9		X	X	X
*Lnpep*	leucyl–cystinyl aminopeptidase	X		X	X
*Lrp8*	low-density lipoprotein receptor-related protein 8		X	X	X
*Ly75*	CD205, CLEC13B		X	X	
*Ly9*	CD229, LY-9	X	X	X	X
*M6pr*	CD222, cation-independent mannose-6-phosphate receptor	X	X	X	X
*Notch2*	NOTCH-2				X
*Pdcd1*	CD279, PD-1	X	X	X	X
*Pdia3*	PDI-A3		X	X	X
*Pdia4*	PDI-A4		X		X
*Pecam1*	CD31, PECAM-1			X	
*Prdx1*	peroxiredoxin-1	X			
*PtprcC*	CD45	X			
*Ptprcap*	CD45-associated protein			X	X
*Ptprj*	CD148			X	X
*Pvr*	CD155, poliovirus receptor	X	X	X	X
*Scarb1*	CD36L1, SCARB-1		X	X	X
*Scarb2*	CD36L2, SCARB-2		X	X	X
*Sell*	CD62L, L-selectin		X		X
*Sema4b*	semaphorin-4B	X	X	X	X
*Sema4c*	semaphorin-4C		X	X	X
*Sema4d*	semaphorin-4D	X	X		
*Slamf1*	CD150, SLAM		X		
*Slc11a2*	divalent cation transporter 1		X		X
*Slc29a1*	equilibrative nucleoside transporter 1		X		
*Slc30a1*	zinc transporter 1		X		
*Slc38a1*	sodium-coupled neutral amino acid transporter 1		X		
*Slc39a10*	zinc transporter ZIP10	X	X	X	X
*Slc39a14*	zinc transporter ZIP14				X
*Slc39a6*	zinc transporter ZIP6	X	X	X	X
*Slc3a2*	CD98, 4F2 heavy chain	X	X	X	X
*Slc7a1*	high-affinity cationic amino acid transporter 1		X		
*Slc7a5*	4F2 light chain	X	X	X	X
*Slc7a6*	Y + L amino acid transporter 2		X		
*Sort1*	sortilin		X		
*Tcirg1*	T cell immune regulator 1			X	
*Tfrc*	CD71, transferrin receptor protein	X	X	X	X
*Tgfb1*	transforming growth factor beta-1		X	X	X
*Thy1*	CD90, Thy-1	X	X	X	X
*Tmx1*	thioredoxin-related transmembrane protein 1			X	X
*Tnfrsf18*	CD357		X		
*Trbv5*	T cell receptor beta chain V region		X	X	X
*Txndc15*	thioredoxin domain-containing protein 15			X	X
*Vdac2*	voltage-dependent anion-selective channel protein 2				X

^a^Identified at an FDR of 4.5 per cent relative to an empirical target decoy database and one unique MPB-modified peptide. The peptide was manually verified from the MS/MS spectrum.

### 3.2. Membranes proteins with labile disulfides are common on T cells

A large repertoire of proteins was identified using the procedure to identify proteins with labile disulfides. The proteins range from activating and inhibitory receptors to cell-adhesion molecules such as integrins, molecules involved in antigen presentation, transporters, and also secreted thiol reductases, and metalloproteinases (tables 2–5; summarized in table 1). These included many of those that we predicted due to the presence of exposed disulfide bonds easily accessible to reducing agents. For example, both partners of the heterodimeric transporter 4F2 and the homodimeric transferrin receptor were found. In both cases, these are known to be disulfide-linked [19,20]. Integrins were commonly observed and there are data indicating that these proteins contain labile disulfides [21]. Several members of the CD2/SLAM family were detected, including CD2, CD244, CD229 and CD150. Many members of this family contain disulfide bonds in addition to the conserved disulfide bond between the sheets of the extracellular immunoglobulin superfamily (IgSF) domain. Enzymes are rare at the leucocyte cell surface [22], but members of the ADAM (‘a disintegrin and metalloproteinase’) family were detected (ADAM10, ADAM15 and ADAM17). CD47 is predicted to contain a labile disulfide that links the IgSF domain with one of the extracellular loops (and an isoform of mouse CD47 has additional extracellular sequence with potential labile Cys residues [23,24]).

**Table 2.** Summary of proteomics data from the reduction and differential Cys labelling of 2B4 cells with TCEP. The Cys residues modified are indicated by residue number (@ followed by residue number in peptide) and whether the modification detected was MPB itself or a hydrolysis derivative (indicated by +H_2_O). Protein probability scores from iProphet meta-searches are shown and where applicable weighted spectral index counts (WSC) are shown for the reduced and control samples, respectively. The percentage sequence coverage indicates the percentage of the protein sequence where peptides were identified. Cys denotes the modified Cysteine number in the protein sequence inclusive of signal peptides.

IPI accession	gene	protein description	protein identification probability	% sequence coverage	WSC control	WSC TCEP reduced	maleimide-modified peptide	modification	Cys
IPI00113869	*Bsg*	CD147, Basigin	1	20.5	1	4			
IPI00112752	*Cd27*	CD27	1	13.6		TCEP only	NCTVTANAECSCSK	MPB+H_2_O@12	106
IPI00223769	*Cd44*	CD44	1	27.4		TCEP only	SQEMVHLVNKEPSETPDQCMTADETR	MPB+H_2_O@19	347
IPI00124830	*Cd47*	CD47	1	9.6		TCEP only	TAFNTDQGSACSYEEEK	MPB+H_2_O@11	142
IPI00123957	*Cd97*	CD97	1	15.7		TCEP only			
IPI00420148	*Env*	GP160	1	36.3		TCEP only	WGCETTGQAYWKPSSSWDLISLK	MPB+H_2_O@3	131
							CNPLVLEFTDAGK	MPB+H_2_O@1	181
							CNPLVLEFTDAGKK	MPB@1	181
							LTLSEVTGQGLCVGAVPK	MPB+H_2_O@12	356
							TFDFYVCPGHTVPTGCGGPR	MPB@16	109
IPI00129526	*Hsp90b1*	endoplasmin	1	17.6	1	13.96	GVVDSDDLPLNVSR		
IPI00133903	*Hspa9*	stress-70 protein	1	61.1	4	47	DQLPADECNK	MPB@8	608
							MEEFKDQLPADECNK	MPB@13	608
							AKCELSSSVQTDINLPYLTMDASGPK	MPB+H_2_O@3	317
IPI00117424	*Icam2*	intercellular adhesion molecule 2	0.9981	5.8		TCEP only			
IPI00119612	*Il2rg*	CD132, cytokine receptor common gamma chain	1			TCEP only	CLQYLVQYR	MPB@1	163
IPI00331413	*Itga6*	integrin alpha 6	1	34.1		TCEP only	FGSCQQGVAATFTK	MPB+H_2_O@4	188
							ACMEETLWLQENIR	MPB+H_2_O@2	562
							SMCGSPSGICLK	MPB@3 MPB+H_2_O@10	489 496
							YQTLNCSVNVR	MPB+H_2_O@6	928
IPI00828582	*Itgal*	integrin alpha-L	1	37.6	1	59.63	GSLLACDPGLSR	MPB+H_2_O@6	108
							RPSSEAEQPCLPGVQFR	MPB+H_2_O@10	1008
							VVVLSSRPVVDVVTELSFSPEEIPVHEVECSYSAR	MPB+H_2_O@30	633
IPI00857195	*Itgav*	integrin alpha-V	1	52.5	1	78.3	ICPLPGTALK	MPB+H_2_O@2	492
							GGQMQCEELVAYLR	MPB+H_2_O@6	565
							ARPVVTVNAGLEVYPSILNQDNKICPLPGTALK	MPB+H_2_O@25	565
							CLQITCQVGR	MPB+H_2_O@1	905
IPI00132474	*Itgb1*	integrin beta-1	1	33.3		TCEP only	FCECDNFNCDR	MPB@4	555
							FQGPTCETCQTCLGVCAEHK	MPB@9	633
IPI00320605	*Itgb2*	Integrin beta-2	1	50		TCEP only	VMASECIQEQSFVIR	MPB@6	421
							VMASECIQEQSFVIR	MPB+H_2_O@6	421
							ALGFTDTVTVQVRPQCECQCR	MPB+H_2_O@16	446
							YNSQVCGGSDR	MPB+H_2_O@6	550
							GHCQCNR	MPB+H_2_O@5	599
							EIFGQYCECDNVNCER	MPB+H_2_O@9	537
IPI00877242	*Itgb3*	integrin beta-3	1	26.6		TCEP only			
IPI00469218	*Lamp1*	lysosome-associated membrane glycoprotein 1	1	20.4		TCEP only			
IPI00312063	*Ldlr*	low-density lipoprotein receptor	0.9995	3.8		TCEP only	TTEDELHICR	MPB+H_2_O@9	843
IPI00119809	*Lgals3bp*	galectin-3-binding protein	1	29.6		TCEP only			
IPI00223987	*Lnpep*	leucyl–cystinyl aminopeptidase	1	49.9		TCEP only	SAFPCFDEPAFK	MPB@5	305
							LPTAIIPLCYELSLHPNLTSMTFR	MPB+H_2_O@9	175
							EPCLHPLEPDEVEYEPR	MPB+H_2_O@3	35
IPI00129646	*Ly9*	CD229, LY-9	1	21.4	1	13	DAEIEHIIWNCPPK	MPB+H_2_O@11	82
IPI00108844	*M6pr*	CD222, cation-independent mannose-6-phosphate receptor	1	21.6		TCEP only			
IPI00125890	*Pdcd1*	CD279, PD-1	1	36.5		TCEP only	QAAFCNGLSQPVQDAR	MPB+H_2_O@5	84
							HEDGHCSWPL	MPB+H_2_O@6	264
							QAAFCNGLSQPVQDAR	MPB@5	84
IPI00121788	*Prdx1*	peroxiredoxin-1	1	16.5		TCEP only			
IPI00126092	*Ptprc*	CD45	1	46.3	9.92	78.43	CQLDNLR	MPB@1	337
							CPDYIIQK	MPB+H_2_O@1	776
							NVINVQTDLGIPETPKPSCGDPAAR	MPB+H_2_O@19	382
							CAEYWPSMEEGTR	MPB@1	749
IPI00177179	*Pvr*	CD155, poliovirus receptor	1	15.4		TCEP only	ENVQYSSVNGDCR	MPB@12	398
IPI00464135	*Sema4b*	semaphorin-4B	1	4		TCEP only	LWVHNGAPVNASASCR	MPB+H_2_O@15	620
IPI00114274	*Sema4d*	semaphorin-4D	1	8.1		TCEP only			
IPI00273801	*Slc39a10*	zinc transporter ZIP10	1	6		TCEP only	CDPEKEAAELPIK	MPB@1	153
IPI00469000	*Slc39a6*	zinc transporter ZIP6	1	10.1		TCEP only	AFCPDLDSDNSGK	MPB+H_2_O@3	153
IPI00114641	*Slc3a2*	CD98, 4F2 heavy chain	1	49.8	5	28			
IPI00331577	*Slc7a5*	4F2 light chain	0.9993	9.8		TCEP only			
IPI00124700	*Tfrc*	CD71, transferrin receptor protein	1	48	2	46	VEQKEECVK	MPB@7	98
IPI00109727	*Thy1*	CD90, Thy-1	1	25.3	1	7	VTSLTACLVNQNLR	MPB+H_2_O@7	28

**Table 3.** Summary of proteomics data from the reduction and differential Cys-labelling of 2B4 cells with Thioredoxin. The modified Cys residues are indicated by residue number (@ followed by residue number in peptide) and whether the modification detected was MPB itself or a hydrolysis derivative (indicated by +H_2_O). Protein probability scores from iProphet meta-searches are shown and where applicable weighted spectral index counts (WSC) are shown for the reduced and control samples, respectively. The percentage sequence coverage indicates the percentage of the protein sequence observed. Cys denotes the modified Cysteine number in the protein sequence inclusive of the signal peptides.

IPI accession	gene	protein description	protein identification probability	% sequence coverage	WSC control	WSC TRX reduced	maleimide-modified peptide	modification	Cys
IPI00123329	*Adam15*	ADAM15	1	13.6		TRX only			
IPI00381630	*Adam17*	ADAM17	1	12.8		TRX only			
IPI00279010	*Bcam*	CD239, BCAM	1	17.6		TRX only			
IPI00113869	*Bsg*	CD147, Basigin	1	46.9	6	30			
IPI00108001	*Cd2*	CD2	1	20.3		TRX only	CEAINPVSK	MPB@1	180
IPI00119703	*Cd244*	CD244, 2B4	1	22.1		TRX only			
IPI00114509	*Cd3d*	CD3 delta	1	26		TRX only			
IPI00223769	*Cd44*	CD44	1	11.5	2	10			
IPI00124830	*Cd47*	CD47	1	14.8	1	11	TAFNTDQGSACSYEEEK	MPB+H_2_O@11	142
IPI00380293	*Cd96*	CD96	1	3.2		TRX only			
IPI00121627	*Clptm1*	cleft lip and palate transmembrane protein 1 homologue	1	19.4	1	21	VAGIFPCPTFK	MPB+H_2_O@7	454
IPI00420148	*Env*	GP160	1	43.9	12.98	190.91	WGCETTGQAYWKPSSSWDLISLK	MPB+H_2_O@3	131
							CNPLVLEFTDAGK	MPB+H_2_O@1	181
							THQALCNTTQK	MPB@6	368
							CNPLVLEFTDAGKK	MPB@1	181
							LTLSEVTGQGLCVGAVPK	MPB+H_2_O@12	356
							TFDFYVCPGHTVPTGCGGPR	MPB@7	100
							EGGLCAALKEECCFYADHTGVVR	MPB+H_2_O@12	533
IPI00126300	*H2-D1*	H-2 class I histocompatibility antigen, D-K alpha chain	1	26.8		TRX only			
IPI00114492	*H2-K1*	H-2 class I histocompatibility antigen, K-B alpha chain	1	29.4		TRX only			
IPI00129526	*Hsp90b1*	endoplasmin	1	58.1	28.94	164.84			
IPI00323357	*Hspa8*	heat shock cognate 71 kDa protein	1	50.5	2.99	41.74			
IPI00880839	*Hspa9*	stress-70 protein	1	75.7	5	288	MEEFKDQLPADECNK	MPB@13	608
							DQLPADECNK	MPB@8	608
							AKCELSSSVQTDINLPYLTMDASGPK	MPB+H_2_O@3	317
							GAVVGIDLGTTNSCVAVMEGK	MPB+H_2_O@14	66
							CELSSSVQTDINLPYLTMDASGPK	MPB@1	317
IPI00129679	*Ifngr1*	CD119, interferon gamma receptor 1	1	5		TRX only	YCISVDGISSFWQVR	MPB+H_2_O@2	223
IPI00321348	*Igsf8*	CD316, immunoglobulin superfamily member 8	1	6.2		TRX only			
IPI00119612	*Il2rg*	CD132, cytokine receptor common subunit gamma	1	30.9		TRX only			
IPI00120155	*Il6st*	CD130, interleukin-6 receptor subunit beta	1	13.2		TRX only			
IPI00132286	*Itgal*	integrin alpha-L	1	49.4	27.88	90.63	GSLLACDPGLSR	MPB+H_2_O@6	108
IPI00120245	*Itgav*	integrin alpha-V	1	9.5	2	7.99			
IPI00132474	*Itgb1*	integrin beta-1	1	37.1		TRX only	LGGIVLPNDGQCHLENNVYTMSHYYDYPSIAHLVQK	MPB+H_2_O@12	299
IPI00877242	*Itgb3*	integrin beta-3	1	7		TRX only			
IPI00310109	*Lamp2*	lysosome-associated membrane glycoprotein 2	1	18.8		TRX only	NLSFWDAPLGSSYMCNK	MPB+H_2_O@15	336
IPI00785217	*Ldlr*	low-density lipoprotein receptor	1	41.3	7.93	83.26	TTEDELHICR	MPB+H_2_O@9	843
IPI00119809	*Lgals3bp*	galectin-3-binding protein	1	34.3	3	32			
IPI00761657	*Lgals8*	galectin-8	1	16.1		TRX only	SSCIVCNTLTQEK	MPB+H_2_O@3	77
IPI00114396	*Lgals9*	galectin-9	1	54	7	77	GMPFELCFLVQR	MPB+H_2_O@7	101
							FEEGGYVVCNTK	MPB+H_2_O@9	73
IPI00606283	*LOC665 506*	TCR chain	1	47.3	1	27.77			
IPI00121600	*Lrp8*	low-density lipoprotein receptor-related protein 8	1	24.1		TRX only			
IPI00129253	*Ly75*	CD205, CLEC13B	1	10.2		TRX only			
IPI00129646	*Ly9*	CD229, LY-9	1	36.3	8	49	DAEIEHIIWNCPPK	MPB+H_2_O@11	82
IPI00108844	*M6pr*	CD222, cation-independent mannose-6-phosphate receptor	1	36		TRX only	AVVMISCNR	MPB+H_2_O@7	146
IPI00125890	*Pdcd1*	CD279, PD-1	1	24	2	17	QAAFCNGLSQPVQDAR	MPB+H_2_O@5	84
IPI00230108	*Pdia3*	PDI-A3	1	33.7	6	23			
IPI00271951	*Pdia4*	PDI-A4	1	7.3		TRX only			
IPI00177179	*Pvr*	CD155, poliovirus receptor	1	12.7		TRX only			
IPI00116921	*Scarb1*	CD36L1, SCARB-1	1	14.4		TRX only	EHSLFLDIHPVTGIPMNCSVK	MPB+H_2_O@18	385
IPI00127447	*Scarb2*	CD36L2, SCARB-2	1	45.6		TRX only	TSLDWWTTDTCNMINGTDGDSFHPLISK	MPB@11	245
IPI00318993	*Sell*	CD62L, L-selectin	1	6.2		TRX only			
IPI00464135	*Sema4b*	semaphorin-4B	1	10.8		TRX only	LWVHNGAPVNASASCR	MPB+H_2_O@15	620
IPI00890869	*Sema4c*	semaphorin-4C	1	6.5		TRX only			
IPI00454115	*Sema4d*	semaphorin-4D	1	20		TRX only			
IPI00131832	*Slamf1*	CD150, SLAM	1	7.1		TRX only			
IPI00315758	*Slc11a2*	divalent cation transporter 1	1	7.7		TRX only	LGVVTGLHLAEVCHR	MPB+H_2_O@13	137
IPI00120769	*Slc29a1*	equilibrative nucleoside transporter 1	1	7.4		TRX only	IVFIPLLMLCNVK	MPB+H_2_O@10	378
IPI00120166	*Slc30a1*	zinc transporter 1	1	4		TRX only			
IPI00459577	*Slc38a1*	sodium-coupled neutral amino acid transporter 1	1	10.8		TRX only	TVYALPTIAFAFVCHPSVLPIYSELK	MPB+H_2_O@14	286
IPI00273801	*Slc39a10*	zinc transporter ZIP10	1	11.5		TRX only			
IPI00114641	*Slc3a2*	CD98, 4F2 heavy chain	1	82.3	38	212			
IPI00121634	*Slc7a1*	high-affinity cationic amino acid transporter 1	1	8.5		TRX only	TPDSNLDQCK	MPB+H_2_O@9	621
IPI00331577	*Slc7a5*	4F2 light chain	1	23.2	0.5	19			
IPI00221632	*Slc7a6*	Y + L amino acid transporter 2	0.9997	9.1		TRX only			
IPI00420955	*Sort1*	sortilin	1	6.7	1	2			
IPI00124700	*Tfrc*	CD71, transferrin receptor protein	1	69.7	30	218	VEQKEECVK	MPB+H_2_O@7	98
							WNIDSSCK	MPB+H_2_O@7	365
IPI00114457	*Tgfb1*	transforming growth factor beta-1	1	35.6		TRX only			
IPI00109727	*Thy1*	CD90, Thy-1	1	17.9		TRX only			
IPI00133834	*Tnfrsf18*	CD357	1	13.5		TRX only			
IPI00122738	*Trbv5*	T cell receptor beta chain V region	1	33.9		TRX only	FIPECPDSSK	MPB+H_2_O@5	86

**Table 4.** Summary of proteomics data from the reduction and differential Cys-labelling of 2B4 cells with PDI. The modified Cys residues are indicated by residue number (@ followed by residue number in peptide) and whether the modification detected was MPB itself or a hydrolysis derivative (indicated by +H_2_O). Protein probability scores from iProphet meta-searches are shown and where applicable weighted spectral index counts (WSC) are shown for the reduced and control samples, respectively. The percentage sequence coverage indicates the percentage of the protein sequence observed. Cys denotes the modified Cysteine number in the protein sequence inclusive of the signal peptides.

IPI accession	gene	protein description	protein identification probability	% sequence coverage	WSC control	WSC PDI reduced	maleimide-modified peptide	modification	Cys
IPI00131881	*Adam10*	ADAM10	1	6.1		PDI only			
IPI00123329	*Adam15*	ADAM15	1	6.9		PDI only			
IPI00762180	*Adam17*	ADAM17	1	16		PDI only			
IPI00113869	*Bsg*	CD147, Basigin	1	43.6	6	38	TQLTCSLNSSGVDIVGHR	MPB+H_2_O@5	157
IPI00108001	*Cd2*	CD2	1	15.1		PDI only	CEAINPVSK	MPB+H_2_O@1	180
IPI00119703	*Cd244*	CD244, 2B4	1	35		PDI only			
IPI00114509	*Cd3d*	CD3 delta	1	13.3		PDI only			
IPI00223769	*Cd44*	CD44	1	11.5	2	10			
IPI00124830	*Cd47*	CD47	1	25.9	1	15	TAFNTDQGSACSYEEEK	MPB+H_2_O@11	142
IPI00380293	*Cd96*	CD96	1	7.1		PDI only	YECIFTLYPEGIK	MPB+H_2_O@3	118
IPI00138061	*Cr1l*	complement regulatory protein Crry	1	9.7		PDI only			
IPI00923031	*EG665 955*	envelope glycoprotein 52	1	33.5	1.50	35			
IPI00420148	*Env*	GP160	1	44.7	12.98	188.41	EECCFYADHTGVVR	MPB+H_2_O@3	533
							EGGLCAALKEECCFYADHTGVVR	MPB+H_2_O@13	534
							THQALCNTTQK	MPB@6	368
							LTLSEVTGQGLCVGAVPK	MPB+H_2_O@12	356
IPI00108870	*Ephb2*	ephrin type-B receptor 2	1	40.6	1	67.73	CGDNVQYAPR	MPB@1	383
							DSGGREDLVYNIICK	MPB+H_2_O@14	370
							NILVNSNLVCK	MPB+H_2_O@10	768
							IEQVIGAGEFGEVCSGHLK	MPB+H_2_O@14	644
IPI00126300	*H2-D1*	H-2 class I histocompatibility antigen, D-K alpha chain	1	24.6		PDI only			
IPI00114492	*H2-K1*	H-2 class I histocompatibility antigen, K-B alpha chain	1	35		PDI only			
IPI00129526	*Hsp90b1*	endoplasmin	1	60.5	28.94	215.59			
IPI00323357	*Hspa8*	heat shock cognate 71 kDa protein	1	47.1	2.99	45.74			
IPI00880839	*Hspa9*	stress-70 protein	1	70	5	277	GAVVGIDLGTTNSCVAVMEGK	MPB+H_2_O@14	66
							AKCELSSSVQTDINLPYLTMDASGPK	MPB+H_2_O@3	317
							MEEFKDQLPADECNK	MPB@13	608
							MEEFKDQLPADECNK	MPB+H_2_O@13	608
							DQLPADECNK	MPB@8	608
IPI00321348	*Igsf8*	CD316, immunoglobulin superfamily member 8	1	6.2		PDI only			
IPI00119612	*Il2rg*	CD132, cytokine receptor common subunit gamma	1	30.9		PDI only	CLQYLVQYR	MPB@1	183
IPI00120155	*Il6st*	CD130, interleukin-6 receptor subunit beta	1	12.9		PDI only			
IPI00266264	*Itgb3*	integrin beta-3	1	6.7		PDI only	NACLPMFGYK	MPB+H_2_O@3	209
IPI00785217	*Ldlr*	low-density lipoprotein receptor	1	40	7.93	85.24	TTEDELHICR	MPB+H_2_O@9	843
IPI00119809	*Lgals3bp*	galectin-3-binding protein	1	32.6	3	30			
IPI00761657	*Lgals8*	galectin-8	1	20.9		PDI only			
IPI00114396	*Lgals9*	galectin-9	1	54	7	90	FEEGGYVVCNTK	MPB+H_2_O@9	73
							GMPFELCFLVQR	MPB+H_2_O@7	101
IPI00223987	*Lnpep*	leucyl–cystinyl aminopeptidase	1	63.3	11	361	SAFPCFDEPAFK	MPB@5	305
							LPTAIIPLCYELSLHPNLTSMTFR	MPB+H_2_O@9	175
IPI00121600	*Lrp8*	low-density lipoprotein receptor-related protein 8	1	17.7		PDI only			
IPI00129253	*Ly75*	CD205, CLEC13B	1	6.5		PDI only			
IPI00129646	*Ly9*	CD229, LY-9	1	35.4	8	40	DAEIEHIIWNCPPK	MPB+H_2_O@11	82
IPI00108844	*M6pr*	CD222, cation-independent mannose-6-phosphate receptor	1	37.4		PDI only	AVVMISCNR	MPB+H_2_O@7	146
							GGDEYDNHCGK	MPB+H_2_O@9	133
IPI00125890	*Pdcd1*	CD279, PD-1	1	24	2	23	QAAFCNGLSQPVQDAR	MPB+H_2_O@5	84
							QAAFCNGLSQPVQDAR	MPB@5	84
IPI00230108	*Pdia3*	PDI-A3	1	42.4	6	36			
IPI00406901	*Pecam1*	CD31, PECAM-1	0.9998	5		PDI only			
IPI00316976	*Ptprcap*	CD45-associated protein	1	20.3		PDI only	CQAEQTR	MPB@1	133
IPI00406609	*Ptprj*	CD148	1	1.9		PDI only			
IPI00177179	*Pvr*	CD155, poliovirus receptor	1	6.9		PDI only			
IPI00116921	*Scarb1*	CD36L1, SCARB-1	1	18.3		PDI only	ESGIQNVSTCR	MPB+H_2_O@10	334
							EHSLFLDIHPVTGIPMNCSVK	MPB+H_2_O@18	385
							CFLFWSGSK	MPB@1	470
IPI00127447	*Scarb2*	CD36L2, SCARB-2	1	50.4		PDI only	TSLDWWTTDTCNMINGTDGDSFHPLISK	MPB@11	245
							DEVLYLFPSDLCR	MPB@12	274
IPI00464135	*Sema4b*	semaphorin-4B	1	7.3		PDI only			
IPI00890869	*Sema4c*	semaphorin-4C	1	6.5		PDI only			
IPI00273801	*Slc39a10*	zinc transporter ZIP10	1	11.4		PDI only			
IPI00469000	*Slc39a6*	zinc transporter ZIP6	1	7.8		PDI only			
IPI00114641	*Slc3a2*	CD98, 4F2 heavy chain	1	82.3	38	223			
IPI00331577	*Slc7a5*	4F2 light chain	1	25	0.50	19.50			
IPI00914724	*Tcirg1*	T cell immune regulator 1	1	5.2		PDI only			
IPI00124700	*Tfrc*	CD71, transferrin receptor protein	1	68.8	30	219	WNIDSSCK	MPB+H_2_O@7	365
IPI00114457	*Tgfb1*	transforming growth factor beta-1	1	34.6		PDI only			
IPI00109727	*Thy1*	CD90, Thy-1	1	37.7		PDI only			
IPI00121341	*Tmx1*	thioredoxin-related transmembrane protein 1	1	29.8	1	16	FIITALPSIYHCK	MPB+H_2_O@12	106
IPI00122738	*Trbv5*	T cell receptor beta chain V region	1	33.9		PDI only			
IPI00378224	*Txndc15*	thioredoxin domain-containing protein 15	1	25.9		PDI only

**Table 5.** Summary of proteomics data from the reduction and differential Cys-labelling of 2B4 cells with GILT reductase. The modified Cys residues are indicated by residue number (@ followed by residue number in peptide) and whether the modification detected was MPB itself or a hydrolysis derivative (indicated by +H_2_O). Protein probability scores from iProphet meta-searches are shown and where applicable weighted spectral index counts (WSC) are shown for the reduced and control samples, respectively. The percentage sequence coverage indicates the percentage of the protein sequence observed. Cys denotes the modified Cysteine number in the protein sequence inclusive of the signal peptides.

IPI accession	gene	protein description	protein identification probability	% sequence coverage	WSC control	WSC TCEP reduced	maleimide-modified peptide	modification	Cys
IPI00381630	*Adam17*	ADAM17	1	11.6		GILT only			
IPI00108001	*Cd2*	CD2	1	20.3		GILT only	CEAINPVSK	MPB@1	180
IPI00119703	*Cd244*	CD244, 2B4	1	22.1		GILT only			
IPI00223769	*Cd44*	CD44	1	11.5	2	8			
IPI00124830	*Cd47*	CD47	1	15.7	1	10	TAFNTDQGSACSYEEEK	MPB+H_2_O@11	142
IPI00380293	*Cd96*	CD96	1	6.8		GILT only			
IPI00121627	*Clptm1*	cleft lip and palate transmembrane protein 1 homologue	1	20.9	1	21			
IPI00138061	*Cr1l*	complement regulatory protein Crry	1	11.6		GILT only			
IPI00111286	*Creld2*	cysteine-rich with EGF-like domain protein 2	1	14		GILT only			
IPI00420148	*Env*	GP160	1	43.1	12.98	152.94	THQALCNTTQK	MPB@6	368
							EECCFYADHTGVVR	MPB+H_2_O@4	533
							CNPLVLEFTDAGK	MPB+H_2_O@1	181
							CNPLVLEFTDAGKK	MPB@1	181
							TFDFYVCPGHTVPTGCGGPR	MPB@7	109
							EGGLCAALKEECCFYADHTGVVR	MPB+H_2_O@12	533
							WGCETTGQAYWKPSSSWDLISLK	MPB@3	131
							LTLSEVTGQGLCVGAVPK	MPB+H_2_O@12	356
IPI00112072	*H13*	minor histocompatibility antigen H13	1	27	3.96	38.57	HAQPALLYLVPACIGFPVLVALAK	MPB@13	326
IPI00126300	*H2-D1*	H-2 class I histocompatibility antigen, D-K alpha chain	1	26.2		GILT only			
IPI00114492	*H2-K1*	H-2 class I histocompatibility antigen, K-B alpha chain	1	32.5		GILT only			
IPI00129526	*Hsp90b1*	endoplasmin	1	58.4	27.94	152.98			
IPI00323357	*Hspa8*	heat shock cognate 71 kDa protein	1	52.8	2.99	50.69			
IPI00880839	*Hspa9*	stress-70 protein	1	67.7	5	256	GAVVGIDLGTTNSCVAVMEGK	MPB+H_2_O@14	66
							CELSSSVQTDINLPYLTMDASGPK	MPB+H_2_O@1	317
							MEEFKDQLPADECNK	MPB@13	608
							AKCELSSSVQTDINLPYLTMDASGPK	MPB+H_2_O@3	317
							DQLPADECNK	MPB@8	608
							AKCELSSSVQTDINLPYLTMDASGPK	MPB@3	317
IPI00990499	*Ifi30*	gamma-interferon-inducible lysosomal thiol reductase	1	35.5		GILT only	VSLYYESLCGACR	MPB+H_2_O@9	69
IPI00129679	*Ifngr1*	CD119, interferon gamma receptor 1	1	5		GILT only			
IPI00119612	*Il2rg*	CD132, cytokine receptor common gamma chain	1			GILT only	CLQYLVQYR	MPB@1	163
IPI00120155	*Il6st*	CD130, interleukin-6 receptor subunit beta	1	10.8		GILT only			
IPI00318012	*Itfg1*	T cell immunomodulatory protein	1	11.1		GILT only			
IPI00120245	*Itgav*	integrin alpha-V	1	6.9	2	6			
IPI00132474	*Itgb1*	integrin beta-1	1	39.8		GILT only			
IPI00266264	*Itgb3*	integrin beta-3	0.9991	2.4		GILT only			
IPI00134549	*Lamp2*	lysosome-associated membrane glycoprotein 2	1	18.8		GILT only	NLSFWDAPLGSSYMCNK	MPB+H_2_O@15	336
IPI00785217	*Ldlr*	low-density lipoprotein receptor	1	41.2	7.93	69.38	TTEDELHICR	MPB+H_2_O@9	843
IPI00119809	*Lgals3bp*	galectin-3-binding protein	1	32.4	3	20			
IPI00761657	*Lgals8*	galectin-8	1	21.2		GILT only			
IPI00114396	*Lgals9*	galectin-9	1	54	7	65	GMPFELCFLVQR	MPB+H_2_O@7	101
							VPYHLVDTIAVSGCLK	MPB+H_2_O@14	138
IPI00223987	*Lnpep*	leucyl–cystinyl aminopeptidase	1	61.2	11	309	SAFPCFDEPAFK	MPB@5	305
							LPTAIIPLCYELSLHPNLTSMTFR	MPB+H_2_O@9	175
IPI00121600	*Lrp8*	low-density lipoprotein receptor-related protein 8	1	26.5		GILT only			
IPI00129646	*Ly9*	CD229, LY-9	1	37.7	8	42	DAEIEHIIWNCPPK	MPB+H_2_O@11	82
IPI00108844	*M6pr*	CD222, cation-independent mannose-6-phosphate receptor	1	39.9		GILT only			
IPI00467908	*Notch2*	NOTCH-2	1	2.6	1	6			
IPI00125890	*Pdcd1*	CD279, PD-1	1	24	2	16			
IPI00230108	*Pdia3*	PDI-A3	1	33.3	6	29			
IPI00271951	*Pdia4*	PDI-A4	1	13.1		GILT only			
IPI00316976	*Ptprcap*	CD45-associated protein	1	20.3		GILT only	CQAEQTR	MPB@1	133
IPI00406609	*Ptprj*	CD148	1	4.5		GILT only			
IPI00177179	*Pvr*	CD155, poliovirus receptor	1	12.7		GILT only			
IPI00116921	*Scarb1*	CD36L1, SCARB-1	0.9995	7.9		GILT only			
IPI00127447	*Scarb2*	CD36L2, SCARB-2	1	47.3		GILT only	DEVLYLFPSDLCR	MPB+H_2_O@12	274
IPI00318993	*Sell*	CD62L, L-selectin	1	9.8		GILT only			
IPI00464135	*Sema4b*	semaphorin-4B	1	7.3		GILT only			
IPI00890869	*Sema4c*	semaphorin-4C	1	6.5		GILT only			
IPI00315758	*Slc11a2*	divalent cation transporter 1	1	10.2		GILT only	LGVVTGLHLAEVCHR	MPB+H_2_O@13	137
IPI00273801	*Slc39a10*	zinc transporter ZIP10	1	10.2		GILT only			
IPI00123428	*Slc39a14*	zinc transporter ZIP14	1	45.7		GILT only			
IPI00469000	*Slc39a6*	zinc transporter ZIP6	1	10.1		GILT only			
IPI00114641	*Slc3a2*	CD98, 4F2 heavy chain	1	68.8	38	194			
IPI00129395	*Slc7a5*	4F2 light chain	1	19.3	0.5	15.5			
IPI00124700	*Tfrc*	CD71, transferrin receptor protein	1	59.1	30	179			
IPI00114457	*Tgfb1*	transforming growth factor beta-1	1	35.6		GILT only			
IPI00109727	*Thy1*	CD90, Thy-1	1	31.5		GILT only			
IPI00121341	*Tmx1*	thioredoxin-related transmembrane protein 1	1	29.8	1	13	FIITALPSIYHCK	MPB+H_2_O@12	106
IPI00122738	*Trbv5*	T cell receptor beta chain V region	0.9998	20.5		GILT only			
IPI00378224	*Txndc15*	thioredoxin domain-containing protein 15	1	16.9		GILT only			
IPI00122547	*Vdac2*	voltage-dependent anion-selective channel protein 2	1	32.9	3	7.99			

### 3.3. Identification of the cysteine residues involved in labile disulfides

The above analysis identified proteins labelled by MPB after reduction, but to work out the structural and functional consequences of each labile disulfide, it is necessary to identify the individual Cys residues that constitute these disulfide bonds. This identification also allows the confirmation that a particular polypeptide contains a labile disulfide bond and has not been co-purified with a biotin-modified protein. To improve the chances of identifying modified peptides, an avidin affinity enrichment step was introduced after trypsin digestion to purify the biotinylated peptides from the tryptic peptide preparation. The MPB-modified peptides were detected in two forms—the second being the maleimide hydrolysis product of MPB. This modified procedure gave increased recognition of MPB-labelled peptides identified from 2B4 cells after reduction with TCEP (table 2), TRX (table 3), PDI (table 4) and GILT (table 5). Only a limited number of Cys residues were detected, indicating high selectivity for labile disulfide bonds. Those Cys not modified were detected by their modification with *N*-acetylamidomethyl from the IAA step prior to trypsin digestion.

**Figure 2.**
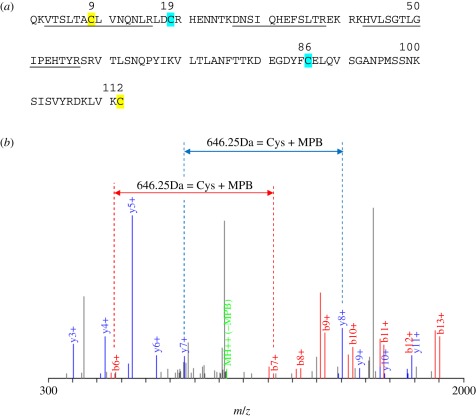
Analysis of Thy-1 isolated after reduction with TCEP showing peptide coverage and MBP-modified peptide. (*a*) Amino acid sequence of mouse Thy-1 showing the peptides identified by mass spectrometry (underlined) and the peptide containing the biotin–maleimide modification (residue 9; yellow), which forms a labile disulfide bond with the Cys (112; yellow) at the C-terminus. Cys (112) would not be expected to be recognized by MS as the predicted tryptic peptide is a single residue that is coupled to the glycophosphatidylinositol anchor. The Cys residues for the other stable disulfide (Cys19 and Cys86) are shown in blue. (*b*). The MS/MS spectrum of peptide VTSLTAC(MPB)LVNQNLR shows good unambiguous coverage of the b^+^ (red peaks) and y^+^ (blue peaks) ion series. Sequential individual amino acid masses were identified in both the b^+^ and y^+^ ions series except for Cys-7, which has the MPB tag attached. A mass difference of 646.25 kDa between b6^+^–b7^+^ (red dashed lines) and y7^+^–y8^+^ (blue dashed lines) corresponds to the mass of Cys + MPB.

The analysis is illustrated for the membrane protein Thy-1 (table 1 and figure 2). The mature Thy-1 protein consists of 112 amino acids with two disulfide bonds. One is the typical disulfide bond found between the beta sheets of IgSF domains, whereas the other is predicted to be at the surface linking the A strand to the C-terminal amino acid of the G strand to which the glycophosphatidylinositol anchor is attached [25]. The total sequence coverage of the mature polypeptide as determined by MS analysis was 36 per cent. Peptides for the predicted inter-sheet disulfide were not covered by the MS analysis, but these inter-sheet disulfide bonds in IgSF domains have a low solvent accessibility and are unlikely to be labile.

There was high specificity for modification of disulfide bonds in the extracellular regions of membrane proteins. Most Cys inside the cell are free sulfhydryls because of the reducing conditions present in the cell. Out of 45 proteins identified with at least one MPB-labelled Cys, only CD45, CD155, CD36L1 and PD-1 had any MPB labels within their cytoplasmic domains, and these were found only with one of the reducing conditions. It is possible that these arise owing to cell death during the labelling giving access to cytosolic Cys residues to the membrane-impermeable MPB.

We have identified an actual labile disulfide bond in approximately 50 per cent of the proteins identified. Not all of the proteins are expressed at the same level on the cell surface and one of the limitations of a proteomics approach is dealing with a large dynamic range of abundances. Therefore, it is possible that we are not detecting MPB-labelled peptides from less abundant proteins on probability grounds. It is also possible that proteins without a labelled peptide may have been co-purified along with a binding partner that did contain an MPB-labelled peptide, and therefore do not contain a labile disulfide bond at all. The purification step included a lectin affinity chromatography step. The number of membrane proteins without glycosylation is relatively few, but these, and those without suitable glycosylation for the lectin, will not be detected. Immunoprecipitation of these molecules under reducing conditions and analysis by mass spectrometry may increase the probability of detecting labile disulfides in these proteins. Finally, the tryptic peptides containing MPB labels might not ionize efficiently in the mass spectrometer, rendering them inert to this screen. Mass spectrometry technology is constantly improving and we predict that more MPB-labelled peptides will be identified in the future.

Generally, the lability of disulfide bonds is dependent on the interplay of a number of factors. First, the disulfide needs to be accessible to the reducing agent; hence, surface disulfide bonds tend to be more labile than buried disulfide bonds. Recent bioinformatics studies that analysed all of the disulfide bonds in the protein data bank based on solvent accessibility, Cα–Cα distance and an estimation of torsion strain on the S–S bonds [1,2] concluded that the most common configuration of the known allosteric disulfide bonds is the –RHStaple. For instance, the allosteric disulfides in the immune co-receptor, CD4, and the HIV envelope protein, gp120, are –RHStaple bonds. A feature of –RHStaple bonds is the close proximity of the α-carbon atoms of the two cysteine residues [26,27]. However, many of the labile disulfide bonds identified in our study were not –RHStaple. This suggests that both bond energetics and solvent accessibility are equally crucial factors in rendering a disulfide bond labile.

### 3.4. Different proteins were identified using various enzymes and chemical-modifying agents

The different reducing conditions all gave proteins with free sulfhydryl groups. The enzymatic treatments gave a wider range of proteins than chemical reduction with TCEP. One might hypothesize that small molecule chemical reductant could ‘access’ and reduce more structurally hindered disulfide bonds than enzymatic reductants, and therefore the proteins identified with enzyme reduction would be a truncated version of the TCEP list. However, this is not observed as PDI, TRX and GILT show a different repertoire of reduced disulfide bonds. There is evidence that enzymes such as TRX can reduce disulfides that have limited solvent-accessibilities and that this is achieved through partial unfolding of the protein domain containing the disulfide bond (e.g. the inter-strand disulfide in domain two of CD4) [28]. This disulfide bond is reduced by TRX secreted by T cells even though the crystal structure [29,30] shows the disulfide to be inward-pointing and totally contained within the core of the tightly folded IgSF domain. Partial unfolding of domain two would be needed to allow access to the active site of TRX and to establish the disulfide-linked homodimer that is the preferred form for the immune co-receptor [31], while the reduced monomer appears to be the preferred receptor for HIV-1 [32]. In the 2B4 hybridoma screens, only three proteins were labelled with MPB on Cys from their inter-sheet IgSF domains: CD2, CD96 and basigin (CD147). All of these were identified with the enzymatic reductants, but none with TCEP reduction, further indicating that some ‘structural’ disulfides may be accessible by enzymes. Interestingly, CD4 was not identified under the screening conditions employed in this study.

### 3.5. Free cysteines are induced by immunological stimuli *in vivo*

There are data to show that extracellular redox potential increases on T cell activation [11] and there is an increase in non-protein thiols at the cell surface following immunization [12], but a key question is whether these changes are sufficient to modify disulfide bonds in membrane proteins. We screened for membrane proteins with free Cys residues following a strong immunological stimulus with LPS given *in vivo* in mice for 3 h, conditions that are known to induce toxic shock and serum GILT accumulation [15,33]. Splenocytes from LPS-treated and control mice were immediately labelled with MPB upon release from the spleen to ensure that the redox state of Cys residues in the proteins was preserved before exogenous oxygen could oxidize reduced disulfide bonds. Cell-surface proteins were purified and subjected to the differential labelling proteomics screen (figure 1) in order to identify proteins that had been reduced as a result of LPS treatment and labelled with MPB. Many labelled proteins were detected after LPS treatment, with relatively few in the control untreated samples. The mass spectrometry data from five separate experiments (12 LPS-treated spleens and 12 control spleens in total) were pooled and analysed using the Oxford Central Proteomics Facility Pipeline, which incorporated normalized spectral index quantitation (SINQ) at the protein level. Thirty-seven proteins were identified (table 6) with at least 10-fold enrichment in the spleens from LPS-treated mice. A diverse range of proteins was identified, including proteins from B cells, T cells and platelets. Proteins involved in B cell activation—CD19, CD22 and CD14, which is a component of the B cell LPS receptor—were identified. Proteins involved in T cell activation and regulation—CD8, SLAM family receptors (SLAM, CRACC, CD84 and Ly-9) and CD132—were identified in activated spleen. Disulfide-reducing enzyme PDI-A1 was also found in LPS-treated spleens. These enzymes have been shown to be present at the cell surface and perform reduction of disulfide bonds [34]. In these experiments, maleimide–biotin-labelled peptides could not be routinely identified by mass spectrometry. This is probably a sensitive issue because of the complex mixture of cell types in spleen, which results in relatively few cells of one type compared with homogeneous cell lines used in the global screens (tables 1–5). However, because the proteins have been purified from the cell lysate using avidin affinity chromatography that involves specific elution with biotin, they must contain, or be associated with, proteins that contain a biotinylated Cys. Many of the proteins identified in the T cell screen (such as integrins) were also identified in this model of inflammation, indicating that modification of membrane glycoproteins by changes in extracellular redox conditions—redox potential and disulfide-modifying enzymes—may be common and affect the activity of many cell-surface proteins.

**Table 6.** Summary of proteomics data from mouse splenocytes that have been activated *in vivo* with LPS and differentially Cys-labelled. The data were filtered to 1% FDR using an empirical target decoy database approach and the protein identifications are at least 10-fold enriched in the LPS spleens relative to control spleens based on SINQ ratios.

IPI accession	gene	protein name	peptides	% coverage	ratio LPS/control
IPI00626485	*Adam9*	ADAM9	2	4.14	LPS only
IPI00113869	*Bsg*	CD147, Basigin	5	22.71	LPS only
IPI00323624	*C3*	complement C3	2	2.71	LPS only
IPI00131091	*C4b*	complement C4-B	5	6.21	LPS only
IPI00308990	*Cd14*	CD14	2	8.74	LPS only
IPI00118168	*Cd163*	CD163	2	2.42	LPS only
IPI00114788	*Cd19*	CD19	2	5.12	LPS only
IPI00108001	*Cd2*	CD2	4	13.08	21.9
IPI00785318	*Cd22*	CD22	12	19.12	10.8
IPI00473824	*Cd244*	CD244, 2B4	2	8.27	LPS only
IPI00129594	*Cd84*	CD84, SLAMF5	2	6.08	LPS only
IPI00110285	*Cd8b1*	CD8 beta	3	15.02	LPS only
IPI00276430	*Clec2d*	CLEC-2d	5	27.54	12.4
IPI00138061	*Cr1l*	complement regulatory protein Crry	5	14.7	40.8
IPI00387418	*Gp5*	GP5	8	23.46	10.6
IPI00129526	*HSP90B1*	endoplasmin	18	25.06	9.9
IPI00308885	*Hspd1*	60 kDa heat-shock protein	5	17.28	22.2
IPI00123342	*Hyou1*	hypoxia-upregulated protein 1	18	27.93	17.3
IPI00122973	*Icam1*	intercellular adhesion molecule 1	3	7.08	LPS only
IPI00109960	*Ighd*	Ig delta chain C region	6	33.07	142.2
IPI00119612	*Il2rg*	CD132, cytokine receptor common subunit gamma	2	6.78	LPS only
IPI00126077	*Itga2*	integrin alpha-2	6	7.3	10.6
IPI00126090	*Itga3*	integrin alpha-3	3	5.13	LPS only
IPI00135010	*Itgax*	integrin alpha-X	6	7.01	13.1
IPI00229516	*Itgb5*	integrin beta-5	2	3.43	13.8
IPI00110508	*Itgb7*	integrin beta-7	3	4.71	LPS only
IPI00408061	*Lgals8*	galectin-8	2	6.99	LPS only
IPI00169585	*Lilrb3*	CD85a, LIR-3	2	4.52	LPS only
IPI00129646	*Ly9*	CD229, LY-9	5	10.09	17
IPI00122815	*P4hb*	PDI-A1	3	11.79	LPS only
IPI00131832	*Slamf1*	CD150, SLAM	4	15.45	LPS only
IPI00128903	*Slamf7*	CD319, CRACC	2	11.67	LPS only
IPI00467600	*Stab2*	stabilin-2	14	7.35	19
IPI00109727	*Thy1*	CD90, Thy-1	3	22.84	LPS only
IPI00320618	*Tlr3*	CD283, toll-like receptor 3	2	3.76	LPS only
IPI00122181	*Tlr7*	toll-like receptor 7	4	4.95	LPS only
IPI00318748	*Tlr9*	CD289, toll-like receptor 9	5	6.88	LPS only

## 4. Discussion

The application of the proteomics screen showed that a large number of leucocyte membrane proteins had labile disulfide bonds that could be reduced by chemical reductants and a variety of enzymes known to be present extracellularly under certain circumstances. The identification of many of these proteins (tables 1–5) and additional ones in the spleens from mice with inflammation induced by LPS (table 6) point to changes in the disulfide bonds of many membrane proteins. This is likely to have significant functional effects. Examples of the effects of labile disulfides are discussed for selected groups of proteins.

A labile disulfide bond was identified in CD132, the common gamma chain of receptors for IL-2, IL-4, IL-7, IL-9 and IL-15 (table 4). There are extensive data indicating that this disulfide bond is important for the activity of these receptors [35]. We analysed this in more detail, showing that mild reducing conditions that break this disulfide bond can affect the activity of this receptor [36]. The presence of CD132 in the LPS experiments suggests that inflammation may affect cytokine receptor activity.

Given the frequency of IgSF domains on membrane proteins of leucocytes, it is not surprising that they are commonly detected [22]. In the example of Thy-1 (figure 2), there are two disulfide bonds—one is the typical disulfide bond found between the beta sheets of IgSF domains, whereas the other was predicted to be at the surface linking the A strand to the final amino acid (of the G strand) to which the glycophosphatidylinositol anchor is attached [25]. Many IgSF domains in leucocyte surface proteins are predicted to have disulfide bonds in addition to the inter-sheet disulfide (e.g. several members of the CD2/SLAM family were identified in the screens including CD2, CD224, CD229 and CD150). Apart from CD229 (discussed above), the precise Cys residues involved are yet to be determined.

**Figure 3.**
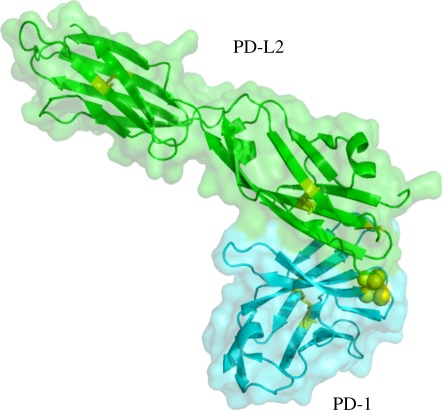
Crystal structure of mouse PD-1 (blue) in complex with mouse PD-L2 (green) extracted from PDB entry 3BP6. Cys 50 (mutated to Ser in the protein used to determine the structure) is shown as yellow spheres and is at the interface of PD-1/PD-L2. Any molecule linked to Cys 50 is likely to interfere with PD-1 binding its ligands.

The majority of Cys residues in the extracellular regions of membrane proteins form disulfide bonds with other Cys residues within the polypeptide or between polypeptides. One interesting exception is PD-1 (CD279), which was detected in all the screens (tables 1–5). PD-1 contains a single IgSF domain and the biotin–maleimide-modified Cys (residue 50) was identified under three reducing conditions (tables 2–4). This residue had been mutated to Ser in the protein used in determining the X-ray crystal structure (PDB; 3BP5) [37]. As labelled Cys 50 was detected only after reduction, it is not present as a free Cys but disulfide-linked to another sulfhydryl group. Biochemical analysis shows that PD-1 is a monomer and hence this residue does not normally cause dimerization [38]. The nature of this interaction is unclear. What is surprising is that this residue is close to the binding site of its ligand (figure 3), and it is possible that some of the PD1 is normally modified in a manner that prevents ligand-binding and that this can be controlled by redox changes that occur during inflammation. However, this cannot occur in humans as there is no free Cys 50 in human PD-1.

Another free Cys was identified in the V-domain of the T cell receptor beta chain. This is not one of the conserved Cys residues but an extra one in this particular V-domain. In some TCR V-domains, a Cys at this position forms a disulfide with an additional Cys in the adjacent strand [39]. The finding that this residue is revealed by reduction suggests that it is disulfide-linked. It should be noted that the finding of a protein in this screen does not imply that all the protein has been modified, but just sufficient levels for detection.

Integrins were among the most common groups of proteins identified in the screens (tables 1–6), and included several alpha and beta chains. Integrins are known to be affected by mutation or reduction of disulfides [5,40], and this indicates that their activity may be modulated by redox changes. For instance, a labile disulfide detected in CD18 (EIFGQYCE*CDNVNCER; table 2) corresponds to the Cys 31 (residue 536) in human CD18, which when mutated and expressed in COS-7 cells gave increased ligand-binding activity [39]. The lifting of constraints by selected disulfides may increase the activity of integrin, and a detailed analysis of labile disulfides in integrins is in progress.

Galectin 1, galectin 8 and galectin 9 were identified. Galectins are cytosolic lectins but can come to the surface and give functional effects [41]. Galectins contain free Cys residues, so it is surprising that they are detected in this screen as any cell-surface galectin should be blocked by the MPM reagent. The finding that Cys residues can be detected raises the possibility that these Cys residues were modified by forming a disulfide bond with either another Cys residue (presumably on another protein) or another adduct that might affect the activity of the galectin in the extracellular environment.

Three members of the ADAM family of metalloproteinases—ADAM9, ADAM15 and ADAM17—were detected in the T cell screen and ADAM9 was also identified from spleen cells; modified Cys were not detected. However, there are data for ADAM17 showing that PDI can cause conformational changes that maintain this enzyme in an inactive state, thus limiting its ability to mediate shedding of cell-surface proteins [42]. This would imply that the activation events discussed here might lead to reduced turnover of cell-surface proteins or proteins in the vicinity via this mechanism, at least through ADAM17, and possibly the other ADAMs.

Members of the scavenger receptor family, CD36L1 and CD26L2, were detected under several reducing conditions. Cys384 in the human CD36L1 has recently been shown to be important in lipid uptake [43]. Both the Cys251 and Cys384 were reported to be free sulfhydryls in CD36L1 [43], whereas in our experiments reducing agent was required before free Cys was detected. It is possible that the culture conditions dictate the status of the disulfide bonds, but these data suggest that the redox state of at least Cys384 may be important in the regulation of lipid uptake.

In some cases, the Cys residue seems unlikely to affect the functional activity. The dimeric state of the transferrin receptor is dependent on two Cys residues (89 and 98 in humans) [19], but surprisingly these disulfide bonds and the dimeric state are not necessary for cell-surface expression and transferrin uptake [44]. The precise labile disulfide bond was not identified in the amino acid transporter system involving disulfide-linked heterodimers with the common CD98 (4F2) chain [45], although it seems likely to be the inter-chain disulfide. It is possible that the generation of free Cys residues is important in forming new associations of cell-surface proteins or affecting their turnover.

The method detected a variety of different types of membrane proteins with labile disulfide bonds, indicating that redox changes during events such as inflammation have broad functional affects. As mentioned above, one cannot rule out proteins being identified on the basis of their association with proteins with labile disulfides, but even concentrating on those proteins where modified Cys-containing peptides have been identified the effects are potentially wide-ranging.

The *in vivo* LPS experiments indicated that many of the proteins identified in the *in vitro* T cell experiments could also be identified under physiological conditions of inflammation. In addition, many other proteins could be identified that were derived from the various cell types in spleen, including B cells, platelets and endothelium, suggesting that a wide variety of cell types could have membrane protein alteration induced by redox changes (note that for these examples the precise Cys residues involved remain to be identified).

## 5. Conclusion

The development of a screening method to detect labile disulfide bonds has demonstrated (i) that they are common in membrane proteins and (ii) that they can be modified during inflammation. This widespread occurrence of labile disulfide bonds in membrane proteins, together with data on the changes in redox potential and secretion of disulfide-altering enzymes, points to a ‘redox-regulator’ mechanism that can give altered membrane protein activity during events such as platelet and immune activation, with implications for their regulation and also events such as virus uptake.

## 6. Experimental procedures

### 6.1. Gamma interferon-inducible lysosomal thiol reductase protein expression and purification

Full-length mouse precursor GILT with an N-terminal 6X His tag behind the signal sequence was cloned into the pFastBac vector (Invitrogen) and expressed in Sf21 insect cells. To purify recombinant protein, cells were pelleted at 1000*g* for 15 min at room temperature and the clarified supernatant was supplemented with 0.5 mM phenylmethylsulfonyl fluoride, 5 mM CaCl_2_, 1 mM NiSO_4_ and 50 mM Tris–Cl (pH 8.0), and stirred at room temperature for 15 min. This solution was then centrifuged at 8000*g* for 15 min at room temperature. The resultant supernatant was filtered and loaded onto TALON beads pre-equilibrated with 20 mM Tris–Cl (pH 8.0), 300 mM NaCl and 10 mM imidazole. Protein was eluted with buffer supplemented with 300 mM imidazole and dialysed into phosphate-buffered saline (PBS) containing 25 µM dithiothreitol (DTT).

### 6.2. Differential labelling of cell lines with thiol-reactive labels

2B4 mouse T cell hybridoma cells (2 × 10^8^) were treated with MPM (2.5 mM in PBS containing 1% bovine serum albumin, BSA) for 30 min at 4°C to label any free Cys on the cell surface. After washing the cells with 3 × 50 ml of 1 per cent BSA in PBS, the cell surface was reduced with either 2.5 mM TCEP, or 0.5 µg ml^−1^ of enzymatic reductant (TRX, PDI or GILT) [17] and 10 µM DTT as a supply of electrons, for 30 min at 25°C. After washing (3 × 50 ml 1% BSA in PBS), the sample was split into two suspensions of 1 × 10^8^ cells. One sample was treated with 2.5 mM MPM for 30 min at 4°C to form a control, and any free Cys formed after reduction in the analyte sample was labelled with 2.5 mM MPB. The cells were washed (3 × 50 ml 1% BSA in PBS) and pelleted for further processing.

### 6.3. Labelling of labile disulfide bonds following inflammation induced by lipopolysaccharide

One microgram of LPS (Sigma Chemical Company, St Louis, MO) in PBS was given intraperitoneally to each adult Balb/c mouse and the spleen taken after 3 h. Control mice received PBS alone. The spleen cells were teased out into RPMI containing 2.5 mM MBP and gently agitated at 4°C for 30 min. The cells were washed with RPMI (3 × 50 ml) and pelleted for further processing. The viability and cell number were comparable between controls and experimental spleens.

### 6.4. Extraction and purification of biotinylated cell-surface glycoproteins

The labelled cell pellets were resuspended in 5 ml lysis buffer (Tris-buffered saline containing 1% Triton X-100 and 5 mM *N*-ethylmaleimide) and rotated at 4°C for 20 min. The cell debris was pelleted at 1600*g* for 30 min and the cell extract was transferred to a clean tube. Lentil lectin beads (300 µl) were added, mixed by rotation overnight at 4°C, washed with 50 ml of wash buffer (TBS containing 0.1% Triton X-100) and pelleted. The cell-surface glycoproteins were eluted from the beads by rotating them in 5 ml of 10 per cent α-methyl glucoside in wash buffer for 4 h at 4°C. The eluant was transferred to a new tube and 300 µl of monomeric avidin beads (Pierce Chemical Company, Northumberland, UK) added, followed by rotation of the mixture overnight at 4°C. The beads were washed with 50 ml of wash buffer and the biotinylated glycoproteins were eluted by rotation in 5 ml of 5 mM biotin in wash buffer for 4 h at 4°C, after which the beads were pelleted and 2.5 ml of the eluant was concentrated into two microcon YM-10 concentrators for in-filter tryptic digest and mass spectrometry.

### 6.5. In-filter PNGase F and trypsin digest

The samples on the filter membranes were washed three times with 200 µl of PBS, spinning the membrane to dryness in-between, then resuspended in 50 µl of PBS to which 6 µl of reaction buffer and 1 µl of PNGase F (New England BioLabs, Ipswich, MA; 500 000 units ml^−1^) were added, incubated overnight at 37°C and spun to dryness on the membrane.

The proteins were digested for mass spectrometry by in-filter digestion of proteins [46]. Briefly, the samples on the filter membranes were denatured by suspending in 8 M urea (500 µl) and incubating at 50°C for 1 h, then washed with 3 × 500 µl aliquots of 25 mM ammonium bicarbonate sample. The proteins were resuspended in 500 µl of reducing buffer (10 mM DTT in 25 mM ammonium bicarbonate) and left at room temperature for 30 min, washed twice with 500 µl of 25 mM ammonium bicarbonate, spinning the membrane to dryness in-between. Alkylating solution of 500 µl (20 mM iodoacetamide in 25 mM ammonium bicarbonate) was added to the sample, incubated in the dark for 1 h and washed twice with 200 µl of 25 mM ammonium bicarbonate, spinning the membrane to dryness in-between. The sample was resuspended in 200 µl 25 mM ammonium bicarbonate and 1 µg trypsin added, and left overnight at 37°C with shaking. The tryptic peptides were eluted through the membrane (3 × 200 µl, 25 mM ammonium bicarbonate) by centrifugation.

### 6.6. Enrichment of maleimide-PEO_2_-biotin-labelled peptides

The pooled eluants containing tryptic peptides were passed over a 50 µl monomeric avidin micro-column. The flowthrough that contained all non-MPB-labelled peptides were collected and evaporated to dryness. MPB-labelled peptides were eluted with acidified acetonitrile (500 µl, 0.4% TFA in 30% acetonitrile) and evaporated to dryness.

### 6.7. LC-mass spectrometry

The tryptic peptide samples were desalted on a C18 packed pipette tip system and injected onto an Ultimate 3000 nano HPLC (Dionex, Sunnyvale, CA) system coupled to an Orbitrap XL mass spectrometer (Thermo Electron, Waltham, MA). Samples were resolved on a 12 cm × 75 µm inner diameter picotip column (New Objective, Woburn, MA), which was packed in-house with ProntoSIL 120-3 C18 ace-EPS (3 micron) phase (Bischoff Chromatography, Leonberg, Germany). Samples were resolved using a 120 min gradient at a flow rate of 300 nl min^−1^. The mass spectrometer was operated in data-dependent acquisition mode, in which 2+, 3+ and 4+ ions were selected for fragmentation. Precursor scans were performed in the Orbitrap at a resolving power of 60 000 (full width half maximum), from which five precursor ions were selected and fragmented in the linear ion trap (‘top-5 method’). Charge state 1+ ions were rejected. Dynamic exclusion was enabled.

### 6.8. Data analysis

RAW data files were converted to the mzXML format using ReAdW (v. 4.2.1) (http://tools.proteomecenter.org/wiki/index.php?title=Software:ReAdW), and submitted to the in-house Central Proteomics Facilities Pipeline (CPFP version) [47], which uses Mascot (Matrix Science, Boston, MA), X!Tandem [48] and OMSSA [49] search engines. Datasets were searched with variable peptide modifications like carbamidomethyl cysteine, oxidized methionine, deamidated asparagine/glutamine, and the appropriate cysteine-modifying label (NEM, MPM or MPB) and maleimide-hydrolysed versions of the labels. Precursor mass tolerance was set at ±20 ppm and MS/MS fragment ion tolerance at ±0.6 Da. Searches were performed against v. 3.64 of the IPI mouse protein sequence database [50].

The resulting peptide identifications from each search engine were validated with PeptideProphet [51]. iProphet was used to combine peptide hits from the three search engines. [52]. ProteinProphet inferred protein identifications from the resulting combined peptide list, and performed grouping of ambiguous identifications [53]. All searches were performed against a concatenated target/decoy database, providing an empirical false discovery rate (FDR) [54] that can be compared with the estimated FDRs from the prophet tools to confirm the validity of results. By default, results are reported at a 1 per cent target/decoy FDR for both peptides and proteins. SINQ [55] at the protein level were performed on grouped datasets to provide quantitative estimates of the relative protein abundance between reduced and control samples. Localization of chemical modifications, when more than one Cys was present in a peptide, was determined by running a ModLS localization algorithm within the CPFP [55].

Protein identifications were exported from the CPFP and uploaded to ProteinCenter (v. 3.7.10003, Proxeon) for filtering, comparison, annotation, classification and interpretation. The 1 per cent FDR filter for identifications calculated within the CPFP was maintained throughout the analysis in ProteinCenter and proteins of interest were restricted to those with at least two unique peptides.

## References

[1] Schmidt B Ho L Hogg PJ. 2006 Allosteric disulfide bonds. Biochemistry 45, 7429–7433 10.1021/bi0603064 (doi:10.1021/bi0603064) 16768438

[2] Hogg PJ. 2009 Contribution of allosteric disulfide bonds to regulation of hemostasis. J. Thromb. Haemost. 7 (Suppl. 1), 13–16 10.1111/j.1538-7836.2009.03364.x (doi:10.1111/j.1538-7836.2009.03364.x) 19630758

[3] Wouters MA George RA Haworth NL. 2007 ‘Forbidden’ disulfides: their role as redox switches. Curr. Protein Peptide Sci. 8, 484–495 10.2174/138920307782411464 (doi:10.2174/138920307782411464) 17979763

[4] Holbrook LM Watkins NA Simmonds AD Jones CI Ouwehand WH Gibbins JM. 2010 Platelets release novel thiol isomerase enzymes which are recruited to the cell surface following activation. Br. J. Haematol. 148, 627–637 10.1111/j.1365-2141.2009.07994.x (doi:10.1111/j.1365-2141.2009.07994.x) 19995400

[5] Chigaev A Zwartz GJ Buranda T Edwards BS Prossnitz ER Sklar LA. 2004 Conformational regulation of α 4 β 1-integrin affinity by reducing agents: ‘inside-out’ signaling is independent of and additive to reduction-regulated integrin activation. J. Biol. Chem. 279, 32435–32443 10.1074/jbc.M404387200 (doi:10.1074/jbc.M404387200) 15166232

[6] Essex DW. 2009 Redox control of platelet function. Antioxid. Redox Signal 11, 1191–1225 10.1089/ARS.2008.2322 (doi:10.1089/ARS.2008.2322) 19061441

[7] Jain S McGinnes LW Morrison TG. 2007 Thiol/disulfide exchange is required for membrane fusion directed by the Newcastle disease virus fusion protein. J. Virol. 81, 2328–2339 10.1128/JVI.01940-06 (doi:10.1128/JVI.01940-06) 17151113 PMC1865930

[8] Ou W Silver J. 2006 Role of protein disulfide isomerase and other thiol-reactive proteins in HIV-1 envelope protein-mediated fusion. Virology 350, 406–417 10.1016/j.virol.2006.01.041 (doi:10.1016/j.virol.2006.01.041) 16507315

[9] Jain S McGinnes LW Morrison TG. 2009 Role of thiol/disulfide exchange in Newcastle disease virus entry. J. Virol. 83, 241–249 10.1128/JVI.01407-08 (doi:10.1128/JVI.01407-08) 18922867 PMC2612340

[10] Schroeder BO 2011 Reduction of disulphide bonds unmasks potent antimicrobial activity of human β-defensin 1. Nature 469, 419–423 10.1038/nature09674 (doi:10.1038/nature09674) 21248850

[11] Angelini G Gardella S Ardy M Ciriolo MR Filomeni G Di Trapani G Clarke F Sitia R Rubartelli A. 2002 Antigen-presenting dendritic cells provide the reducing extracellular microenvironment required for T lymphocyte activation. Proc. Natl Acad. Sci. USA 99, 1491–1496 10.1073/pnas.022630299 (doi:10.1073/pnas.022630299) 11792859 PMC122218

[12] Castellani P Angelini G Delfino L Matucci A Rubartelli A. 2008 The thiol redox state of lymphoid organs is modified by immunization: role of different immune cell populations. Eur. J. Immunol. 38, 2419–2425 10.1002/eji.200838439 (doi:10.1002/eji.200838439) 18792398

[13] Lawrence DA Song R Weber P. 1996 Surface thiols of human lymphocytes and their changes after *in vitro* and *in vivo* activation. J. Leuk. Biol. 60, 611–618 10.1002/jlb.60.5.611 8929552

[14] Schwertassek U Balmer Y Gutscher M Weingarten L Preuss M Engelhard J Winkler M Dick TP. 2007 Selective redox regulation of cytokine receptor signaling by extracellular thioredoxin-1. EMBO J. 26, 3086–3097 10.1038/sj.emboj.7601746 (doi:10.1038/sj.emboj.7601746) 17557078 PMC1914094

[15] Lackman RL Jamieson AM Griffith JM Geuze H Cresswell P. 2007 Innate immune recognition triggers secretion of lysosomal enzymes by macrophages. Traffic 8, 1179–1189 10.1111/j.1600-0854.2007.00600.x (doi:10.1111/j.1600-0854.2007.00600.x) 17555533

[16] Lackman RL Cresswell P. 2006 Exposure of the promonocytic cell line THP-1 to *Escherichia coli* induces IFN-γ-inducible lysosomal thiol reductase expression by inflammatory cytokines. J. Immunol. 177, 4833–4840 16982925 10.4049/jimmunol.177.7.4833

[17] Hastings KT Cresswell P. 2011 Disulfide reduction in the endocytic pathway: immunological functions of gamma-interferon-inducible lysosomal thiol reductase. Antioxid. Redox Signal 15, 657–668 10.1089/ars.2010.3684 (doi:10.1089/ars.2010.3684) 21506690 PMC3125571

[18] Clarkson NG Brown MH. 2009 Inhibition and activation by CD244 depends on CD2 and phospholipase C-γ1. J. Biol. Chem. 284, 24 725–24 734 10.1074/jbc.M109.028209 (doi:10.1074/jbc.M109.028209) PMC2757176 19586919

[19] Jing SQ Trowbridge IS. 1987 Identification of the intermolecular disulfide bonds of the human transferrin receptor and its lipid-attachment site. EMBO J. 6, 327–331 3582362 10.1002/j.1460-2075.1987.tb04758.x PMC553399

[20] Wagner CA Lang F Broer S. 2001 Function and structure of heterodimeric amino acid transporters. Am. J. Physiol. Cell Physiol. 281, C1077–C1093 11546643 10.1152/ajpcell.2001.281.4.C1077

[21] Chigaev A Waller A Zwartz GJ Buranda T Sklar LA. 2007 Regulation of cell adhesion by affinity and conformational unbending of α4β1 integrin. J. Immunol. 178, 6828–6839 (doi:178/11/6828[pii]) 17513731 10.4049/jimmunol.178.11.6828

[22] Barclay AN Brown MH Law SKA McKnight AJ Tomlinson MG van der Merwe PA. 1997 Leucocyte antigens facts book, 2nd edn. London, UK: Academic Press

[23] Hatherley D Graham SC Turner J Harlos K Stuart DI Barclay AN. 2008 Paired receptor specificity explained by structures of signal regulatory proteins alone and complexed with CD47. Mol. Cell 31, 266–277 10.1016/j.molcel.2008.05.026 (doi:10.1016/j.molcel.2008.05.026) 18657508

[24] Rebres RA Vaz LE Green JM Brown EJ. 2001 Normal ligand binding and signaling by CD47 (integrin-associated protein) requires a long range disulfide bond between the extracellular and membrane-spanning domains. J. Biol. Chem. 276, 34 607–34 616 10.1074/jbc.M106107200 (doi:10.1074/jbc.M106107200) 11454874

[25] Williams AF Gagnon J. 1982 Neuronal cell Thy-1 glycoprotein: homology with immunoglobulin. Science 216, 696–703 10.1126/science.6177036 (doi:10.1126/science.6177036) 6177036

[26] Katz BA Kossiakoff A. 1986 The crystallographically determined structures of atypical strained disulfides engineered into subtilisin. J. Biol. Chem. 261, 15 480–15 485 3096989

[27] Wells JA Powers DB. 1986 *In vivo* formation and stability of engineered disulfide bonds in subtilisin. J. Biol. Chem. 261, 6564–6570 3516996

[28] Matthias LJ Yam PT Jiang XM Vandegraaff N Li P Poumbourios P Donoghue N Hogg PJ. 2002 Disulfide exchange in domain 2 of CD4 is required for entry of HIV-1. Nat. Immunol. 3, 727–732 10.1038/ni815 (doi:10.1038/ni815) 12089508

[29] Ryu SE 1990 Crystal structure of an HIV-binding recombinant fragment of human CD4. Nature 348, 419–426 10.1038/348419a0 (doi:10.1038/348419a0) 2247146 PMC5638305

[30] Wang JH 1990 Atomic structure of a fragment of human CD4 containing two immunoglobulin-like domains. Nature 348, 411–418 10.1038/348411a0 (doi:10.1038/348411a0) 1701030

[31] Maekawa A Schmidt B Fazekas de St Groth B Sanejouand YH Hogg PJ. 2006 Evidence for a domain-swapped CD4 dimer as the coreceptor for binding to class II MHC. J. Immunol. 176, 6873–6878 16709847 10.4049/jimmunol.176.11.6873

[32] Matthias LJ Azimi I Tabrett CA Hogg PJ. 2010 Reduced monomeric CD4 is the preferred receptor for HIV. J. Biol. Chem. 285, 40 793–40 799 10.1074/jbc.M110.190579 (doi:10.1074/jbc.M110.190579) PMC3003380 20974843

[33] Hamerman JA Tchao NK Lowell CA Lanier LL. 2005 Enhanced Toll-like receptor responses in the absence of signaling adaptor DAP12. Nat. Immunol. 6, 579–586 10.1038/ni1204 (doi:10.1038/ni1204) 15895090 PMC1282462

[34] Popescu NI Lupu C Lupu F. 2010 Extracellular protein disulfide isomerase regulates coagulation on endothelial cells through modulation of phosphatidylserine exposure. Blood 116, 993–1001 10.1182/blood-2009-10-249607 (doi:10.1182/blood-2009-10-249607) 20448108 PMC2924232

[35] Leonard WJ. 2001 Cytokines and immunodeficiency diseases. Nat. Rev. Immunol. 1, 200–208 10.1038/35105066 (doi:10.1038/35105066) 11905829

[36] Metcalfe C Cresswell P Barclay AN In preparation Interleukin-2 signalling is modulated by a labile disulfide bond in CD132 chain of the receptor. 10.1098/rsob.110036 PMC3352089 22645657

[37] Lazar-Molnar E Yan Q Cao E Ramagopal U Nathenson SG Almo SC. 2008 Crystal structure of the complex between programmed death-1 (PD-1) and its ligand PD-L2. Proc. Natl Acad. Sci. USA 105, 10 483–10 488 10.1073/pnas.0804453105 (doi:10.1073/pnas.0804453105) PMC2492495 18641123

[38] Zhang X 2004 Structural and functional analysis of the costimulatory receptor programmed death-1. Immunity 20, 337–347 10.1016/S1074-7613(04)00051-2 (doi:S1074761304000512[pii]) 15030777

[39] Newell EW Ely LK Kruse AC Reay PA Rodriguez SN Lin AE Kuhns MS Garcia KC Davis MM. 2011 Structural basis of specificity and cross-reactivity in T cell receptors specific for cytochrome c-I-E(k). J. Immunol. 186, 5823–5832 10.4049/jimmunol.1100197 (doi:10.4049/jimmunol.1100197) 21490152 PMC3754796

[40] Nolan SM Mathew EC Scarth SL Al-Shamkhani A Law SK. 2000 The effects of cysteine to alanine mutations of CD18 on the expression and adhesion of the CD11/CD18 integrins. FEBS Lett. 486, 89–92 (doi:S0014-5793(00)02247-X[pii]) 11113444 10.1016/s0014-5793(00)02247-x

[41] Bi S Earl LA Jacobs L Baum LG. 2008 Structural features of galectin-9 and galectin-1 that determine distinct T cell death pathways. J. Biol. Chem. 283, 12 248–12 258 10.1074/jbc.M800523200 (doi:10.1074/jbc.M800523200) PMC2431002 18258591

[42] Willems SH Tape CJ Stanley PL Taylor NA Mills IG Neal DE McCafferty J Murphy G. 2010 Thiol isomerases negatively regulate the cellular shedding activity of ADAM17. Biochem. J. 428, 439–450 10.1042/BJ20100179 (doi:10.1042/BJ20100179) 20345372

[43] Yu M Romer KA Nieland TJ Xu S Saenz-Vash V Penman M Yesilaltay A Carr SA Krieger M. 2011 Exoplasmic cysteine Cys384 of the HDL receptor SR-BI is critical for its sensitivity to a small-molecule inhibitor and normal lipid transport activity. Proc. Natl Acad. Sci. USA 108, 12 243–12 248 10.1073/pnas.1109078108 (doi:10.1073/pnas.1109078108) 21746906 PMC3145699

[44] Alvarez E Girones N Davis RJ. 1989 Intermolecular disulfide bonds are not required for the expression of the dimeric state and functional activity of the transferrin receptor. EMBO J. 8, 2231–2240 2507316 10.1002/j.1460-2075.1989.tb08347.x PMC401153

[45] Wagner CA Broer A Albers A Gamper N Lang F Broer S. 2000 The heterodimeric amino acid transporter 4F2hc/LAT1 is associated in *Xenopus* oocytes with a non-selective cation channel that is regulated by the serine/threonine kinase sgk-1. J. Physiol. 526 Pt 1, 35–46 10.1111/j.1469-7793.2000.00035.x (doi:10.1111/j.1469-7793.2000.00035.x) 10878097 PMC2269991

[46] Wisniewski JR Zielinska DF Mann M. 2011 Comparison of ultrafiltration units for proteomic and N-glycoproteomic analysis by the filter-aided sample preparation method. Anal. Biochem. 410, 307–309 10.1016/j.ab.2010.12.004 (doi:10.1016/j.ab.2010.12.004) 21144814

[47] Trudgian DC Thomas B McGowan SJ Kessler BM Salek M Acuto O. 2010 CPFP: a central proteomics facilities pipeline. Bioinformatics 26, 1131–1132 10.1093/bioinformatics/btq081 (doi:10.1093/bioinformatics/btq081) 20189941

[48] MacLean B Eng JK Beavis RC McIntosh M. 2006 General framework for developing and evaluating database scoring algorithms using the TANDEM search engine. Bioinformatics 22, 2830–2832 10.1093/bioinformatics/btl379 (doi:10.1093/bioinformatics/btl379) 16877754

[49] Geer LY Markey SP Kowalak JA Wagner L Xu M Maynard DM Yang X Shi W Bryant SH. 2004 Open mass spectrometry search algorithm. J. Proteome Res. 3, 958–964 10.1021/pr0499491 (doi:10.1021/pr0499491) 15473683

[50] Kersey PJ Duarte J Williams A Karavidopoulou Y Birney E Apweiler R. 2004 The International Protein Index: an integrated database for proteomics experiments. Proteomics 4, 1985–1988 10.1002/pmic.200300721 (doi:10.1002/pmic.200300721) 15221759

[51] Keller A Nesvizhskii AI Kolker E Aebersold R. 2002 Empirical statistical model to estimate the accuracy of peptide identifications made by MS/MS and database search. Anal. Chem. 74, 5383–5392 10.1021/ac025747h (doi:10.1021/ac025747h) 12403597

[52] Shteynberg D Deutsch EW Lam H Eng JK Sun Z Tasman N Mendoza L Moritz RL Aebersold R Nesvizhskii AI. 2011 iProphet: Multi-level integrative analysis of shotgun proteomic data improves peptide and protein identification rates and error estimates. Mol. Cell. Proteomics. 10.1074/mcp.M111.007690 (doi:10.1074/mcp.M111.007690) PMC3237071 21876204

[53] Nesvizhskii AI Keller A Kolker E Aebersold R. 2003 A statistical model for identifying proteins by tandem mass spectrometry. Anal. Chem. 75, 4646–4658 10.1021/ac0341261 (doi:10.1021/ac0341261) 14632076

[54] Elias JE Gygi SP. 2007 Target-decoy search strategy for increased confidence in large-scale protein identifications by mass spectrometry. Nat. Meth. 4, 207–214 10.1038/nmeth1019 (doi:10.1038/nmeth1019) 17327847

[55] Trudgian DC Ridlova G Fischer R Mackeen MM Ternette N Acuto O Kessler BM Thomas B. 2011 Comparative evaluation of label-free SINQ normalized spectral index quantitation in the central proteomics facilities pipeline. Proteomics 11, 2790–2797 10.1002/pmic.201000800 (doi:10.1002/pmic.201000800) 21656681

